# Volatile Semiochemicals Emitted by *Beauveria bassiana* Modulate Larval Feeding Behavior and Food Choice Preference in *Spodoptera frugiperda* (Lepidoptera: Noctuidae)

**DOI:** 10.3390/jof10060438

**Published:** 2024-06-20

**Authors:** Arturo Ramírez-Ordorica, Sandra Goretti Adame-Garnica, Hilda Eréndira Ramos-Aboites, Robert Winkler, Lourdes Macías-Rodríguez

**Affiliations:** 1 Instituto de Investigaciones Químico Biológicas, Universidad Michoacana de San Nicolás de Hidalgo, Edificio B3, Ciudad Universitaria, Morelia C.P. 58030, Mexico; 1211542f@umich.mx; 2 Laboratorio de Análisis Bioquímico e Instrumental, Unidad de Genómica Avanzada, Cinvestav, Km 9.6, Libramiento Norte, Carretera Irapuato-León, Irapuato C.P. 36824, Mexico; hilda.ramos@cinvestav.mx (H.E.R.-A.); robert.winkler@cinvestav.mx (R.W.)

**Keywords:** semiochemicals, fungal volatiles, fusel alcohols, insect behavior, olfactometry, UPLC-MS

## Abstract

*Beauveria bassiana* is an entomopathogenic fungus that parasitizes and kills insects. The role of volatile organic compounds (VOCs) emitted by *B. bassiana* acting as semiochemicals during its interaction with lepidopterans is poorly explored. Here, we studied the effect of VOCs from *B. bassiana* and 3-methylbutanol (as a single compound) on the feeding behavior of L2 larvae of *Spodoptera frugiperda* in sorghum plants. Additionally, we assessed whether fungal VOCs induce chemical modifications in the plants that affect larval food preferences. Metabolomic profiling of plant tissues was performed by mass spectrometry and bioassays in a dual-choice olfactometer. The results showed that the larval feeding behavior was affected by the *B. bassiana* strain AI2, showing that the insect response is strain-specific. Furthermore, 80 µg of 3-methylbutanol affected the number of bites. The larval feeding choice was dependent on the background context. Fragment spectra and a matching precursor ion mass of 165.882 *m*/*z* enabled the putative identification of 4-coumaric acid in sorghum leaves exposed to fungal VOCs, which may be associated with larval deterrent responses. These results provide valuable insights into the bipartite interaction of *B. bassiana* with lepidopterans through VOC emission, with the plant as a mediator of the interaction.

## 1. Introduction

Microbial volatile organic compounds (VOCs) are low-molecular-weight molecules that easily vaporize at environmental temperatures and are commonly produced and emitted by microbes. Numerous studies have discussed the importance of these microbial VOCs as signaling molecules for intra- and inter-kingdom interactions in nature [1]. For example, microbial VOCs elicit plant biochemical changes that improve their tolerance to various abiotic and biotic stresses [2,3,4]. Understanding the evolutionary significance of these communicative phenomena mediated by volatile molecules is one of the principal goals in chemical ecology.

*Beauveria bassiana* is a dimorphic and facultative fungus with a cosmopolitan distribution that specializes in parasitizing and consuming various groups of arthropods, including insects [5]. The infection of the insect host occurs through the adhesion and germination of hydrophobic spores, followed by the penetration of the germ tube into the external cuticle. The action of fungal toxins and mechanical damage caused by the internal fungal mycelium growth lead to the insect’s death [6]. Notably, fungal entomopathogens can interact with their insect hosts without physical contact through the emission of VOCs, which act as “semiochemicals” eliciting physiological or behavioral responses in responding individuals [1,2,3]. For instance, arthropods can respond to the presence of entomopathogenic fungi by avoiding them in many cases [7] and, in other cases, by exhibiting an attractive response [8,9].

Different strains of *B. bassiana* emit VOCs with varied functional and structural complexity [10,11,12]. Nevertheless, the most abundant compounds belong to the alcohol family. The biosynthesis of aromatic and branched-chain alcohols, referred to as fusel alcohols, is common in many fungal species. These alcohols are derived from amino acids via the Ehrlich pathway [13,14] and have been extensively studied as biomarkers of fungal presence in fermented food sources [14,15,16]. However, many insect taxa are highly sensitive to these fusel alcohols [17], and they are often used as bait for field experiments [18,19]. Particularly, 3-methylbutanol, derived from L-leucine [20], attracted lepidopterans in field trapping experiments [21]. 

*B. bassiana* also exhibits a symbiotic lifestyle thriving as an endophyte of plants. The fungus improves iron nutrition and induces plant defense responses against phytopathogens [22,23,24]. The parasitic and symbiotic lifestyles of *B. bassiana* have raised new questions about the interaction between the fungus and insects with plants acting as important mediators [10,25,26,27,28]. Additionally, the role of volatile molecules in this multitrophic interaction remains unclear. 

Lepidopterans are insect herbivores highly sensitive to aromas, which exert physiological and behavioral effects on moths at very low concentrations. *Spodoptera littoralis* can respond to 10^−14^ g of geraniol and linalool, which are floral terpenoid alcohols produced by plants [29]. This remarkable sensitivity is due to insects’ environmental perception being closely linked to their chemoreception capacities. As a result, aromas trigger different and complex behaviors adapted to their changing environment and the aromatic context [30]. Many insect behavioral choices, such as food source location, nesting site selection, mate searching, or avoidance of competitors and harmful agents, depend on VOC perception [31,32,33,34]. In insect herbivores, such as lepidopteran larvae, plant aromas are used as selection markers, inducing both attraction and repellent effects [35,36].

The moth *Spodoptera frugiperda* is a polyphagous pest native to North America. Since 2017, it has rapidly expanded to various regions of the world, particularly in Africa, Asia, and Oceania. This expansion has led to significant damage and losses, with particular concern in poor and developing countries [37,38,39]. This lepidopteran demonstrates high adaptability to diverse environments upon introduction, and it shows rapid development of resistance to various synthetic insecticides commonly used for its control [40,41,42].

Ramírez-Ordorica et al. [11] reported that VOCs from *B. bassiana* alter the reproductive behavior of *S. frugiperda*. In particular, the oviposition differed according to the virulence of the strains, indicating that the adult response is specific to the fungal VOC profile. Moreover, 3-methylbutanol was found to affect egg-laying behavior, demonstrating that this compound acts as a semiochemical that modifies the oviposition behavior in *S. frugiperda*. 

The host plant selection for consumption constitutes a crucial decision in an insect’s life, as food quality profoundly influences its performance and fecundity. Under this scenario, the perception of VOCs emitted by fungal entomopathogens may affect the insect’s feeding behaviors and food choices. Thus, this study aimed to answer the following questions: (i) Can VOCs emitted by *B. bassiana* evoke an innate behavioral response in the larvae of *S. frugiperda*? (ii) Can plants previously exposed to fungal volatiles lead to an aversion response in the insect? (iii) Can fungal volatiles induce chemical modifications in plants? (iv) How do insects respond to 3-methylbutanol in terms of feeding and food choice? (v) What is the chemical response of plants to 3-methylbutanol exposure? This study endeavors to provide insights into the effect of volatile chemical signals emitted by *B. bassiana* on lepidopterans, utilizing plants as mediators of the interaction. 

## 2. Materials and Methods

### 2.1. Spodoptera frugiperda L2 Rearing

The L2 instar of *S. frugiperda* were maintained in plastic containers under controlled conditions in a growth chamber (Lumistell^®^, Guanajuato, Mexico; relative humidity of 25%, 28 °C, 16 h light/8 h darkness photoperiod). Larvae were fed with an artificial diet as described by Serra-Ruíz et al. [43]. Adults were kept in paper bags and fed with a 15% honey solution. The sex ratio during the assays was maintained close to 1:1.

### 2.2. Maintenance of Beauveria bassiana

The *B. bassiana* AI2 strain was isolated from a cadaver of *Phyllophaga* sp. exhibiting signs of mycosis, while *B. bassiana* AS5 was isolated from soil cores collected in Chihuahua, Mexico [11]. The fungal VOCs’ profiles were previously identified by Ramírez-Ordorica et al. [11]. Both strains emit the fusel alcohol 3-methylbutanol as the most abundant compound, with its abundance in AI2 being 53.4% and in AS5 being 72.73%. The fungal strains were cultured on potato dextrose agar medium (PDA, BD Bioxon^®^, Mexico City, Mexico) and maintained in darkness for ~15 d. Subsequently, spores were harvested from mature mycelium by scraping the medium and rinsing with 2 mL of a 0.1% Tween solution. The spore suspension was adjusted to a density of 10^6^ spores mL^−1^ by counting them in a Neubauer chamber and stored at 4 °C for no more than 24 h before use.

### 2.3. Planting and Cultivation of Sorghum bicolor

Seeds of *S. bicolor* were sequentially rinsed in 12% sodium hypochlorite (CLORALEX^®^, Mexico city, Mexico), 70% ethanol (Alymel^®^, Mexico city, Mexico), and sterile water, with agitation for 3 min each. Subsequently, the seeds were germinated in Petri dishes containing agar-water medium (10% *v*/*v*) and grown in a growth chamber at 25% relative humidity, 28 °C, 16 h light/8 h darkness photoperiod for 4 d until the root length reached ~1 cm. Finally, the seedlings were transplanted into ~100 mL of 0.25× Murashige and Skoog (MS) medium (1 g basal salts, Sigma^®^, St. Louis, MO, USA; 6 g sucrose, BD Bioxon^®^, Mexico city, Mexico; 10 g phytoagar, PhytoTech^®^, Lenexa, KS, USA; 1 L water, adjusted to pH 6) in a 500 mL volume glass container. Each container housed three plants, which were allowed to grow for 7 d, preventing the plants from touching the container’s walls. Under these conditions, each plant developed a phenotype with three leaves.

### 2.4. Establishment of the Plant and Fungus Interaction System through Volatile Emission

**Figure 1:**
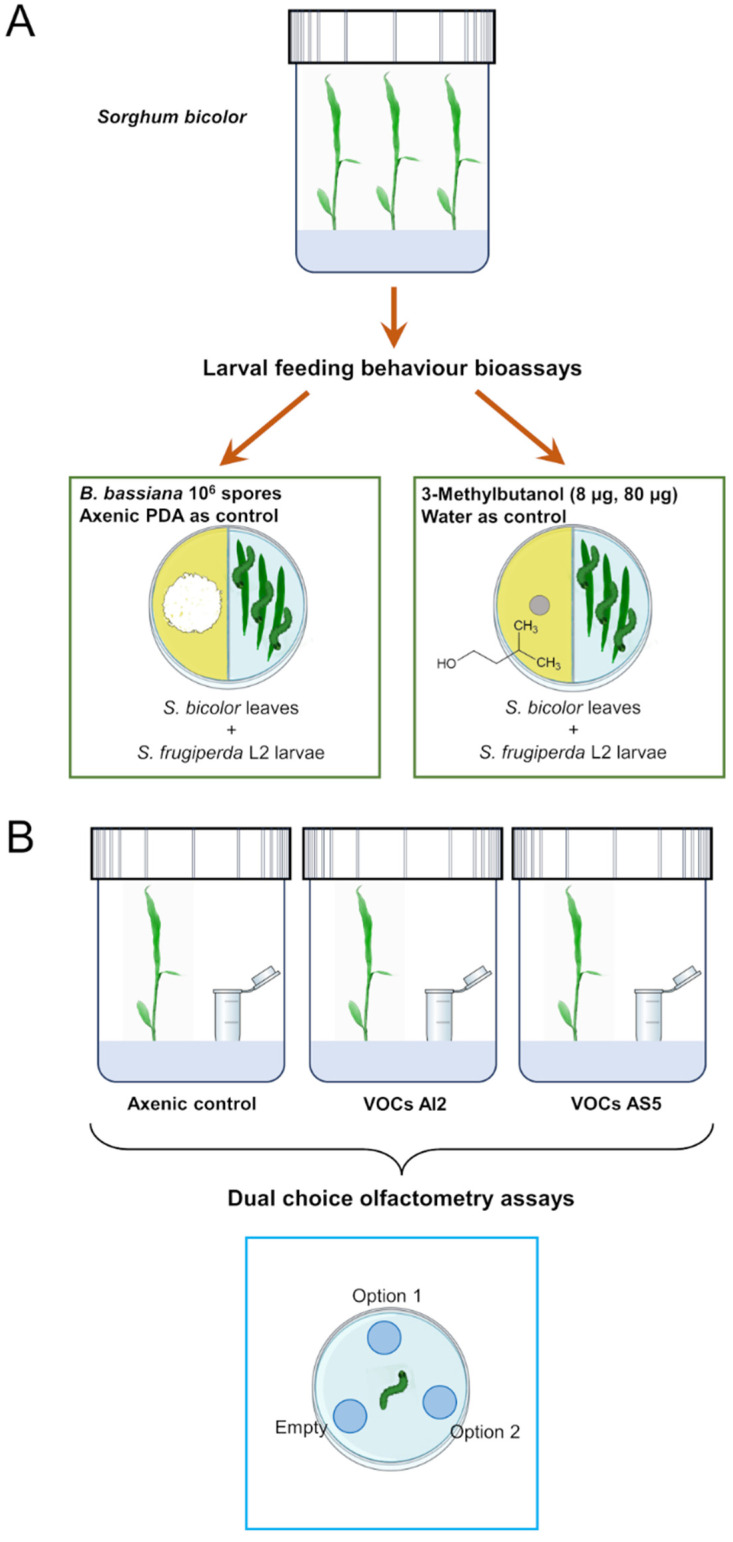
Experimental strategy used to investigate the effect of volatiles emitted by *Beauveria bassiana* on the larval feeding behavior (**A**) and (**B**) food choice preference in *Spodoptera frugiperda*.

The plant–fungus volatile interaction system consisted of introducing a 4 mL glass vial with 2 mL PDA culture medium slant inoculated with 10^6^ spores mL^−1^ inside the glass container. These treatments will be referred to as VOCs AI2 and VOCs AS5 (Figure 1A). The control treatment consisted of an axenic vial with PDA medium. The plants were grown under the same conditions mentioned above. After 7 d of the interaction, plants from all treatments were harvested. Subsequently, the foliage was removed and separated for subsequent biological and metabolomic profile assays.

### 2.5. In Vitro Bioassays to Determine the Effect of B. bassiana VOCs on Larval Herbivory

Divided I-Petri dishes were used to study the effect of fungal VOCs on the larval feeding behavior (Figure 1B). On one side of the I-Petri dish, 10^6^ spores of the AI2 or AS5 strains were inoculated onto PDA culture medium. The fungus was grown for 8 d in the growth chamber. After this time, three fresh leaves of *S. bicolor* and three L2 larvae of *S. frugiperda* were simultaneously placed on the other side of the I-Petri dish. Larvae were previously isolated and fasted for ~5 h. I-Petri dishes were then placed in the growth chamber, allowing the larvae to feed ad libitum for 12 h. Subsequently, the foliage was collected and scanned to measure the leaf area, the number of insect bites on the leaf, and the total area removed by the larvae through the ImageJ^®^ software (https://imagej.net/ij/, accessed on 19 May 2023). The missing leaf tissue was calculated and expressed as a percentage of herbivory. I-Petri dishes with PDA medium without fungal inoculation were used as controls. Each repetition consisted of a minimum of 38 leaves and insects. The experiments were performed in triplicates (n = 114–138 leaves). 

### 2.6. Trials with 3-Methylbutanol

Divided I-Petri dishes were used to study the effect of 3-methylbutanol (8 and 80 μg, >98.5%, Sigma-Aldrich^®^ I9392, St. Louis, MO, USA) on the larval feeding behavior (Figure 1A). The doses were chosen according to Ramírez-Ordorica et al. [11]. On one half of divided I-Petri dishes, a 5 mm paper disc saturated with either 8 μg or 80 μg of 3-methylbutanol was placed over the PDA medium, while on the other half of the dish, three leaves and three L2 *S. frugiperda* larvae were simultaneously placed. As a control, 1 μL of deionized water was used. These I-Petri dishes were then placed in the growth chamber, allowing the larvae to feed ad libitum for 12 h (Figure 1). Subsequently, the foliage was collected and scanned to measure the leaf area, the number of insect bites on the leaf, and the total area removed. Each repetition consisted of a minimum of 19 leaves per triplicate (n = 57–59 leaves).

### 2.7. Insect Olfactometry Bioassays

A three-compartment olfactometer device was constructed for food choice bioassays, based on the design by Kecskeméti et al. [44], using plastic Petri dishes and 0.6 mL Eppendorf tubes (Corning^®^, Glendale, AZ, USA) (Figure 1B). Three holes were drilled in the bottom of the Petri dish, arranged equidistantly between them, and Eppendorf tubes were attached with silicone to each hole. Each device was cleaned using compressed air (Steren^®^, Mexico City, Mexico) and allowed to ventilate for a period of 72 h before use. The following pairs of treatments were arranged in the olfactometer devices: distilled water (200 μL) vs. artificial diet (180 mg), 3-methylbutanol (8 μg) vs. artificial diet, 3-methylbutanol vs. distilled water, distilled water vs. artificial diet, uninoculated PDA medium vs. PDA medium with fungal mycelium (AI2 or AS5), unexposed sorghum leaf vs. leaf exposed to fungal VOCs (VOCs AI2 or VOCs AS5), and empty vs. empty as an absolute control. In all cases, one Eppendorf tube remained empty for contrast, and no larvae were observed inside the empty tube. 

One L2 larva of *S. frugiperda*, previously fasted for 5 h, was placed in the center of each device. The devices were then kept in darkness for a period of 12 h. After this time, the frequency of larval response in the olfactometer was observed. A response was recorded when the larva fell into the Eppendorf tube or remained on the edge of the Eppendorf tube after the time elapsed. The absence of these behaviors was considered as no response. A total of 10–21 insects per repetition were used, and experiments were conducted in triplicate (n = 30–63 insects).

### 2.8. Analysis of Metabolites from S. bicolor Foliage by Ultra-Performance Liquid Chromatography-Mass Spectrometry (UPLC-MS) 

The metabolite profiles of control plants and those exposed to VOCs from AI2 and AS5 were conducted in an Accela UPLC system (Thermo^®^, Waltham, MA, USA) with a C18 Hypersil Gold column (Thermo^®^) maintained at 25 °C, coupled to an LCQFleet mass spectrometer (Thermo^®^). To determine whether the fusel alcohol modulates the plant metabolome, a group of plants were exposed to 8 μg of pure 3-methylbutanol. In this treatment, the compound was placed in an Eppendorf vial. Foliar tissues from all treatments were previously lyophilized, ground using liquid nitrogen, and dissolved in 500 μL of acidified extraction buffer (aqueous solution of 75% methanol, HPLC grade, Fermont@, Monterrey, Nuevo Leon and 0.1% formic acid, Sigma^®^). The samples were then stored at −20 °C for 72 h and centrifuged (Eppendorf^®^, Hamburg, Germany) at 15,000 rpm for 10 min at 4 °C. Finally, the supernatant was filtered (Whatman^®^, Maidstone, UK, 0.2 μm) for subsequent analysis. The samples were analyzed using a mobile phase consisting of an aqueous solution of 0.1% formic acid (solvent A) and 75% MeOH plus 0.1% formic acid (solvent B), with a capillary temperature of 280 °C, capillary voltage of 33 V, and acquiring 3 microscans per second. The chromatographic program consisted of 90% solvent A at 0 min, 50% at 7 min, and 0% at 20 min, returning to initial conditions at 25 min with a flow rate of 400 μL min^−1^, and an injection volume of 4 μL. For the tentative identification of the ions 165.882 and 437.402 *m*/*z*, we used a direct infusion electrospray ionization and mass spectrometry (DIESI-MS/MS), with ten scans for each fragmentation condition. MS/MS fragmentation was achieved by collision-induced dissociation (CID) in LCQFleet mass spectrometer, using nitrogen as neutral collision gas with an energy of 17–18 eV per assay. MS/MS data and the average signal intensity of mass spectra from the ten scans were used for comparative searching with GNPS MASST server (https://masst.gnps2.org/, accesed on 17 May 2024). In addition, the tentative identification was double-checked by comparing the ion mass fragmentation pattern with that reported in published literature [45,46,47].

### 2.9. Data Analysis and Statistics

The data obtained from herbivory were analyzed by using generalized linear models (glm) fitted to a gamma distribution using R language v 4.3.0 (R Core Team) [11]. The frequencies obtained by olfactometry were compared using a Chi-square test. All experiments were conducted in triplicate, with a significance level of 0.05. The raw data generated by mass spectrometry were converted to .mzML format using the MSConvert module of (v 3.0.23240-b4f8aed) ProteoWizard [48] and analyzed with MZmine 3 software (v 3.9.0) [49]. This analysis involved signal detection, chromatogram construction, and signal alignment across all files. In total, a matrix containing 8685 signals specific to foliage extracts was generated. Signals present in at least 70% of the analyzed sample files were retained, and zero values were imputed in the matrix with the average ion intensity across all treatments. This resulted in 116 ions (retention time and *m*/*z* signals in two-dimensional data) for analysis using the random forest (RF) algorithm with 500 trees to investigate their contribution to the model. Finally, a total of 47 ions from the original matrix were retained by discarding those whose signals appeared in the blanks. This generated matrix was analyzed by using principal component analysis (PCA), and the first two resulting principal components were plotted with ~74.4% of the total variance retained in ordinated data. The significance of patterns in the distribution of ordered data was tested using PERMANOVA test with 999 permutations. 

## 3. Results

### 3.1. The Foliar Herbivory and the Degree of Damage Caused by the Chewing Insect S. frugiperda Is Influenced by the Type of fungal VOCs It Perceives

**Figure 2:**
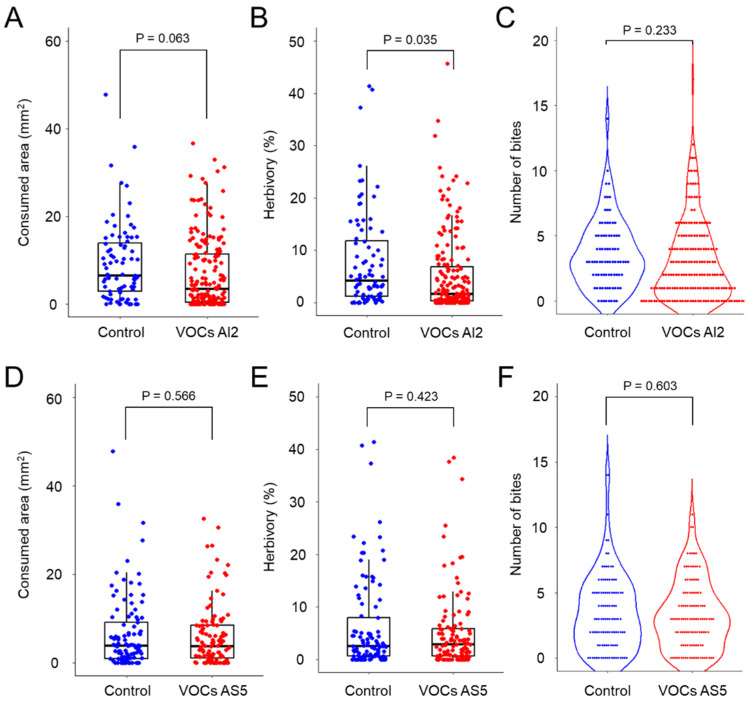
*Spodoptera frugiperda* larval feeding behavior in response to volatiles emitted by the AI2 (**A**–**C**) and AS5 (**D**–**F**) strains of *Beauveria bassiana*. Panels (**A**,**D**) correspond to the feeding area (mm^2^) (removed tissue after 12 h of consumption). Panels (**B**,**E**) show the percentage of herbivory (missing leaf area with respect to the total leaf area). Panels (**C**,**F**) correspond to the number of bites per leaf inflicted by larvae. Control refers to treatments with uninoculated PDA culture medium. n = 114–183, glm with gamma distribution, α = 0.05.

To investigate whether the presence of mycelia from *B. bassiana* affects the larval feeding behavior, we performed an experimental design by using I-Petri plates (Figure 1). The results showed that the average feeding area decreased when larvae perceived the pool of VOCs emitted by AI2, although this decrease was not statistically significant (Figure 2A). Similarly, there was a tendency for the number of bites to decrease (Figure 2C). Notably, herbivory decreased by ~40% (*p* = 0.035) (Figure 2B). Moreover, VOCs from AS5 did not affect herbivory, and the degree of foliar damage was similar to that observed in the control treatment (Figure 2D–F). These findings suggest that insect feeding behavior is dependent on the VOC profile emitted by specific fungal strains.

### 3.2. Context-Dependent Deterrent Effects of VOCs from AI2 and AS5

**Figure 3:**
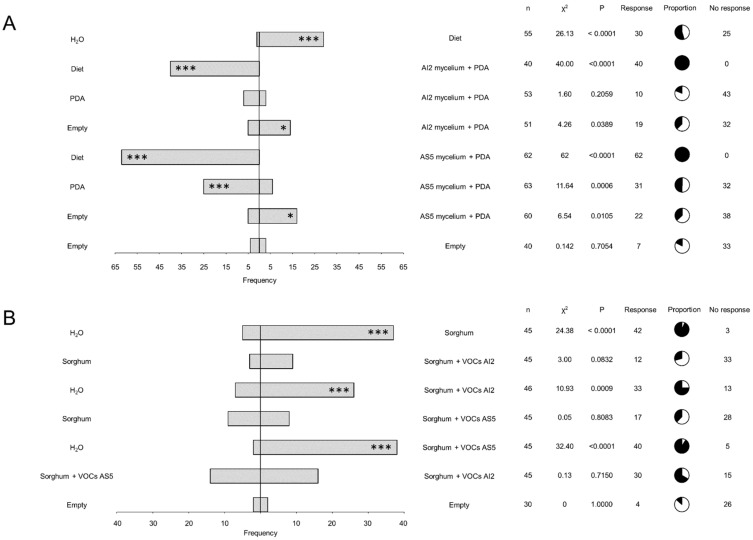
Larval dual-choice olfactometry bioassays in response to pairs of odor sources. (**A**) The bioassays utilized mycelium from the strains AI2 and AS5 cultured in PDA culture medium. n = 40–63. (**B**) Bioassays were performed on sorghum foliage unexposed and exposed to VOCs from the strains AI2 and AS5. n = 30–46. The bars indicate the frequency of insects that selected either odor source. Pie charts show proportions of responding (black) and not responding (white) individuals. Statistical significance according to Chi-square two-sides test (* *p* < 0.05, *** *p* < 0.001) at α = 0.05.

We used different pairs of treatments in the dual-food choice system to determine whether the fungal VOCs act as deterrents of L2 larvae of *S. frugiperda*. These treatments included the PDA culture medium, mycelium from *B. bassiana* AI2 and AS5 grown in PDA, water, artificial diet, and empty tube (Figure 3A). The results showed a consistent food choice response in larvae, regardless of the presence of mycelium. Larvae predominately chose the artificial diet when it was included as an option. Furthermore, PDA was more frequently chosen by the insect over *B. bassiana* AI2 or AS5 mycelia + PDA. This response was notably significant (*p* = 0.0006) in the treatment PDA vs. AS5 mycelium + PDA treatment. However, when the larvae lacked an alternative food option, they chose the treatments containing AI2 mycelium + PDA or AS5 + PDA medium.

### 3.3. The Exposure of Sorghum to Fungal VOCs Did Not Affect the Larval Food Choice

To determine the role of sorghum plants as mediators of communication between fungal entomopathogens and insects via VOC emission, sorghum seedlings were exposed to fungal VOCs emitted by AI2 and AS5 strains. Subsequently, the sorghum foliage was collected and used for food choice experiments. The results obtained from the dual-choice olfactometer showed a clear preference for sorghum foliage, regardless of whether it was exposed to fungal volatiles or not (Figure 3B). Furthermore, no significant differences were observed in larval preferences to sorghum + VOCs AS5 or sorghum + VOCs AI2. However, a higher frequency was observed for sorghum + VOCs AS5 compared to sorghum + VOCs AI2 when the water was included as a food choice option. 

### 3.4. 3-Methylbutanol Does Not Affect Food Choice Behavior but May Influence the Sorghum Foliage Consumption

**Figure 4:**
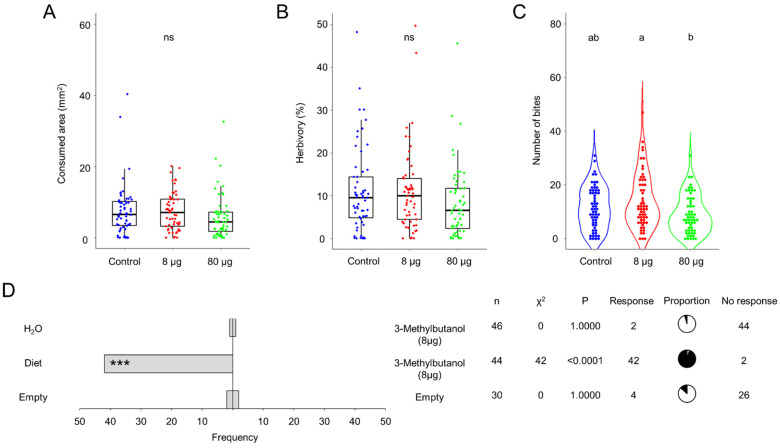
Larval feeding behavior of L2 larvae of *Spodoptera frugiperda* on *Sorghum bicolor* foliage exposed to 8 and 80 µg of 3-methylbutanol. (**A**) Feeding area (mm^2^) (removed tissue after 12 h of consumption). (**B**) Percentage of herbivory (missing leaf area with respect to the total leaf area). (**C**) Number of bites per leaf inflicted by larva. n = 114–183, analyzed with glm with gamma distribution, α = 0.05. Different letters indicate statiscally significant differences, ns means not statiscally significant. (**D**) Olfactometry dual-choice bioassay of L2 larvae exposed to 8 μg of 3-methylbutanol compared to water and artificial diet. The bars indicate the frequency of insects that selected either odor source. Pie charts show proportions of responding (black) and not responding (white) individuals. Statistical significance according to Chi-square two-sides test (*** *p* < 0.001) at α = 0.05.

The predominant volatile emitted by AI2 and AS5 strains is 3-methylbutanol [11]. Consequently, we studied the effect of two doses of this alcohol, 8 and 80 µg, on the larval feeding and food choice behaviors (Figure 4A–D). The results suggest that the effect of 3-methylbutanol on foliage consumption is dose-dependent. The average consumed area and herbivory were reduced with the 80 µg dose, although this result was not statistically significant (Figure 4A,B). In particular, the 80 µg dose diminished the average of number of bites compared to the 8 µg treatment (Figure 4C). Meanwhile, olfactometry results showed that the 8 µg dose of 3-methylbutanol had a deterrent effect on larvae, as the insects did not respond to the presence of the fungal alcohol or water (Figure 4D). Moreover, when 3-methylbutanol was present, the insects were able to perceive and choose the artificial diet (*p* ˂ 0.0001). Thus, 3-methylbutanol may play a crucial role in altering the larval feeding behavior of *S. frugiperda* during the bipartite interaction with *B. bassiana*, probably through a deterrence mechanism.

### 3.5. VOCs Emitted by B. bassiana Induce the Accumulation of Phenolic Metabolites in S. bicolor

**Figure 5:**
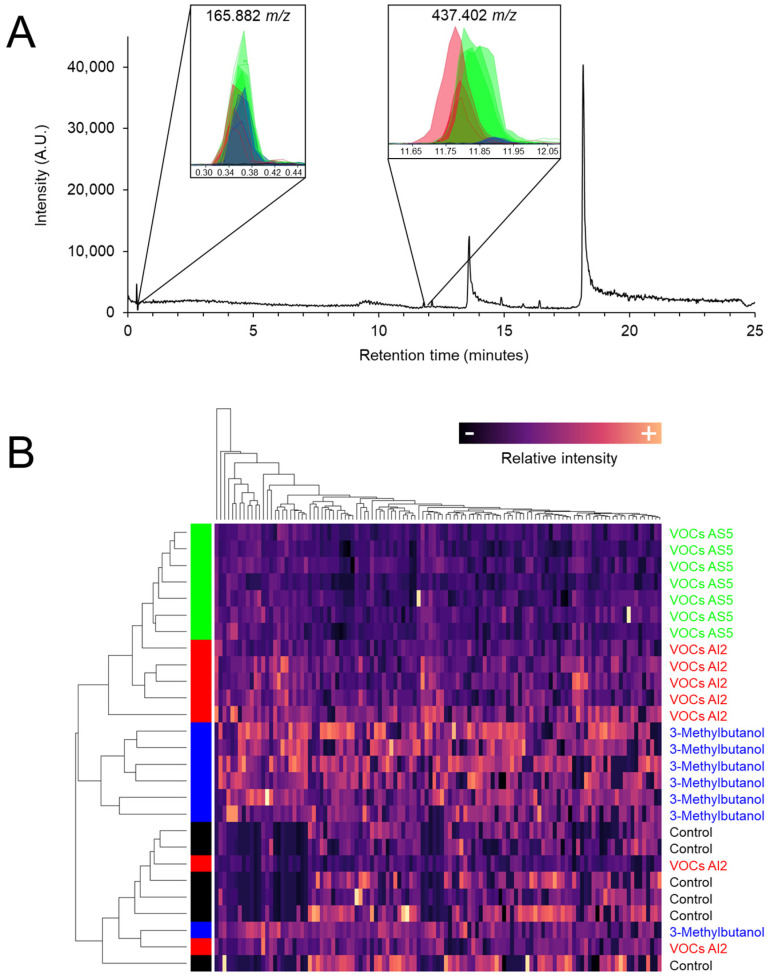
(**A**) Representative chromatogram of the chemical composition of sorghum foliage exposed to VOCs emitted by *B. bassiana*. Insets correspond to the abundance of the ion signals 165.882, 353.347, and 437.402 *m*/*z*. Green, red, blue, and black colors indicate VOCs AS5, VOCs AI2, 3-methylbutanol, and control treatments, respectively. (**B**) Heatmap of the normalized signal intensities of the 116 ions obtained from the metabolic profiles. Hierarchical clustering with Euclidean distance.

*S. bicolor* plants were exposed to VOCs emitted by *B. bassiana* strains AI2 and AS5, as well as to 8 μg of the fungal alcohol 3-methylbutanol. The metabolomic profiling of foliar tissue obtained by UPLC-MS showed that the fungal VOCs induced global changes in ion intensities, particularly in ions 165.882 and 437.402 *m*/*z* (Figure 5A). Notably, 3-methylbutanol induced the greatest variation in the signal intensity of ion 437.402 *m*/*z*. Interestingly, the plant samples from VOCs AS5 treatment were grouped in a separate branch in the heatmap (Figure 5B). 

**Figure 6:**
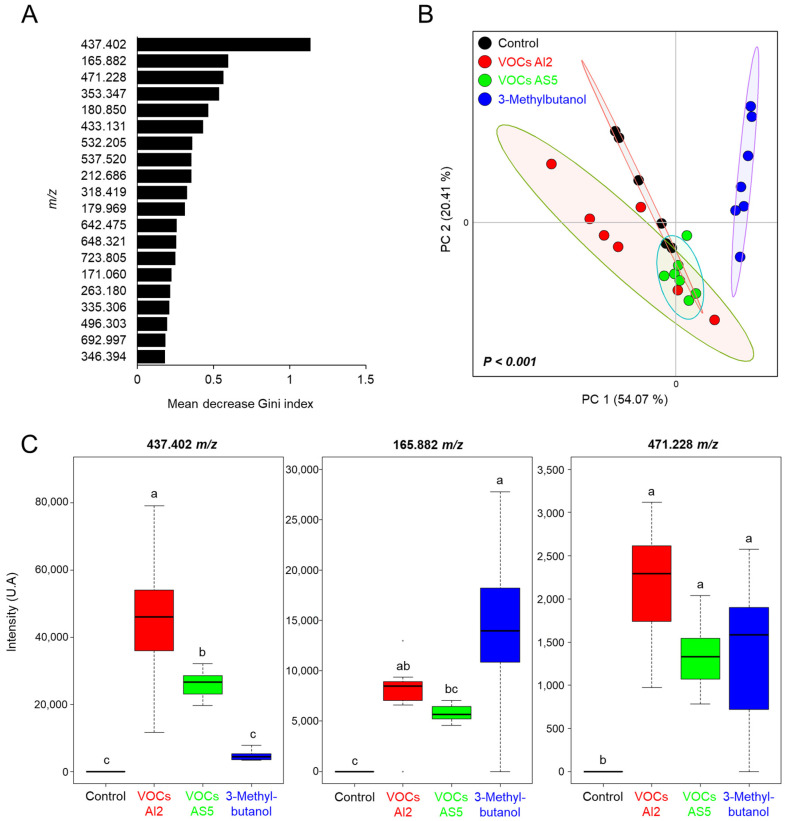
(**A**) Biomarker ions obtained by random forest model for differentiating between the *Sorghum bicolor* foliage treatments, including exposure to VOCs AI2, VOCs AS5, and 8 µg of 3-methylbutanol. Random forest with 500 trees. (**B**) Principal component analysis from the chemical profiles of sorghum plants differentially treated with the fungal VOCs. PERMANOVA test, α = 0.05. (**C**) Mean intensities from the ion signals with the highest Gini index, n = 6. Different letters indicate statiscally significant differences by one-way ANOVA, Tukey post hoc, α = 0.05.

The ions selected by the RF algorithm were within the range of 165.882–692.997 *m*/*z*, with the ion 437.402 *m*/*z* showing the highest contribution to model classification between the treatment categories (Figure 6A). The ordination plot clearly delineates a distinct region corresponding to the 3-methylbutanol treatment. In contrast, the control, VOCs AS5, and VOCs AI2 treatments were grouped together, indicating their similarity (Figure 6B). The abundances of the ion signals 165.882, 437.402, and 471.228 *m*/*z*, which had the highest Gini index, are shown in Figure 6C. The ion 437.402 *m*/*z* exhibited significant variation among the treatments, with its abundance increasing in VOCs AI2 and VOCs AS5 treatments compared to the control, but showing the highest intensity in plants exposed to VOCs from AI2. Otherwise, the ion 165.882 *m*/*z* increased in plants exposed to both VOCs from AI2 and 3-methylbutanol. Conversely, the ion 471.228 *m*/*z* did not show significant differences in abundance among the VOCs AI2, VOCs AS5, and 3-methylbutanol treatments, but it consistently differed from the control. These results indicate that the plant response to fungal VOCs is strain-specific and that 3-methylbutanol shows distinct activity as a single compound.

The mass spectra fragmentation pattern obtained by DIESI MS/MS of the closer parent ion 164.980 *m*/*z* allowed the putative identification of 4-coumaric acid (Table 1). According to the existing literature, the fragment ions 119.05 [M+H-CO]^+^ and 147.04 [M+H-H_2_O]^+^ *m*/*z* are typical for this compound [45]. The fragmentation patterns of the ion 437.275 *m*/*z* did not match with any compound in the database (Table 1). Interestingly, Kazuno et al. [46] and Llorent-Martínez et al. [47] reported the ion 437.275 *m*/*z* to be consistent with *C*-glycosidic flavonoids such as dihydrochalcone glycosides. This ion remains mainly as a [M+H]^+^ ion, and its fragmentation produces minor ions such as 419 *m*/*z* [M+H-H_2_O]^+^. However, its putative identification will require further analysis.

## 4. Discussion

The plant host selection by insect herbivores is crucial for comprehending the co-evolutionary and selective pressures that shape chemical communication and biotic interactions in natural ecosystems [50]. Plants and insects are exposed to complex blends of VOCs, including those emitted by microorganisms, which significantly affect both organisms [1,51]. Fungal entomopathogens, natural enemies of insects, have been reported to manipulate various insect taxa across different life cycle contexts [8,9,26,52]. However, the extent to which VOCs emitted by these specialized microbes modulate the feeding behavior and food choice in Lepidoptera remains unclear. In this study, we used second-instar larvae of *S. frugiperda*, which begin to make holes in leaves. Our results suggest that VOCs emitted by the entomopathogenic fungus *B. bassiana* can induce behavioral changes in the feeding habits of the larvae. This opens up the possibility that microbial entomopathogens in nature may commonly manipulate insect hosts through volatile semiochemicals. 

Notably, herbivory rates are altered in insects infected by phytopathogenic microbes [53], leading to compensatory feeding behaviors. For example, Lepidoptera larvae regulate their protein–carbohydrate intake during baculovirus infection, exhibiting selectivity in their food choices. Similarly, locusts increase their carbohydrate consumption when infected with *Metarhizium acridum* [54,55,56]. Various insect taxa can detect fungal entomopathogens and actively avoid them by moving away from the source of VOC emission [57,58,59]. The results from our study show that the larvae of *S. frugiperda* recognize the presence of *B. bassiana* upon perceiving the VOCs emitted by the mycelium, leading to modifications in their feeding activity. Furthermore, the insect response was strain-specific, and the compound 3-methylbutanol may partly account for the alteration in larval feeding behavior, as evidenced by a reduction in the number of bites observed with a high dose of the compound. At least three theories could explain this finding: (1) Evasion: fungal VOCs induce an innate evasion response in the insect. (2) Confusion: fungal compounds saturate the insect’s olfactory systems, causing confusion and interfering with proper leaf odor recognition. (3) Deterrence: fungal VOCs trigger a deterrent behavior, imparting an unpleasant taste or rendering the leaves unpalatable. These scenarios are likely compatible and contribute to the observed appetitive response in the insect. 

Lepidopteran olfactory perception varies depending on the insect’s developmental stage, transitioning from the larval phase to the primarily nectarivorous adult instar, aligning with the trophic niche utilized by the insect. The shift in VOC perception likely reflects the insect’s adaptation to distinct ecological roles and dietary preferences across different life stages [60]. Notably, the response of *S. frugiperda* larvae L2 was influenced by the pathogenicity profile of isolates AI2 and AS5. Herbivory was negatively affected in L2 larvae exposed to the fungal VOCs emitted by *B. bassiana* strain AI2, whereas the treatment with the AS5 strain’s VOCs did not affect larval feeding behavior. Previous studies have indicated that the strain AI2 exhibits greater pathogenicity than AS5, and quantitative rather than qualitative differences can be observed between their VOC profiles [11]. This result suggests that larvae may differentially respond to fungal source pathogenicity, highlighting the importance of considering isolate-specific effects in further studies. The exact nature of the molecules implicated in the larval-specific response remains unknown, but it is plausible to speculate that chemical information regarding isolate virulence is encoded by the pool of VOCs, which can be perceived by the insect’s neuro-olfactory system, thereby affecting feeding behavioral responses in distinct ways. Interestingly, the insect’s perception of 3-methylbutanol, the main compound emitted by AI2 and AS5, did not elicit the same behavioral response as observed in the VOCs AI2 treatment. This is consistent with the hypothesis that a complex blend of odors elicits different behavioral responses than an individual compound [30,61]. However, 3-methylbutanol plays a crucial role for the fungus in inducing the yeast-to-mycelium transition [62]. Furthermore, the compound exhibits a fungistatic effect against phytopathogens [63] and acts as a semiochemical in various insect families such as Nymphalidae, Noctuidae, Geometridae, Drepanidae, and Pylaridae [21,64]. 

Insect herbivores perceive numerous odors in their environment, providing crucial information about the presence of plants, competitors, and potential threats such as predators and pathogens [33]. To discriminate between multiple aromatic sources, their olfactory response must be adaptive and responsive to changing environmental contexts [65,66,67]. The results from olfactory bioassays conducted with *S. frugiperda* in this study revealed a higher frequency of choosing PDA medium or artificial diet over *B. bassiana* mycelium. This food choice behavior may be interpreted as a deterrent response to the presence of mycelium. Interestingly, when the insect lacks an alternative food source, it opts to consume the PDA with mycelium, indicating an effort to obtain sustenance under severe restriction conditions. Conversely, the compound 3-methylbutanol did not affect the larval food choice behavior, indicating that the insect’s response to fungal odors is primarily driven by the blend of fungal VOCs. Thus, this study contributes to our understanding of the role of VOCs emitted by infectious agents present in food sources, triggering a deterrent response in insects and reducing the likelihood of these populations falling ill [68,69]. Undoubtedly, in an open environment, numerous other factors such as other larval stages, exposition time, the physicochemical properties of the aromatic signal, the specificity of insect receptors to these molecules, and the quality and intensity of the VOC emission may also impact insect behavior [33]. 

Plants play a crucial role as important mediators in the interaction between *B. bassiana* and their insect hosts [10,15,16,20,21]. Our study revealed that the fungal pool of VOCs induces subtle modifications in the chemical profile of the leaves, as evidenced by the larval food choice bioassays; however, despite these modifications, the odor of the leaves remained unchanged. Notably, our mass spectrometry analysis allowed for the putative identification of the phenylpropanoid 4-coumaric acid, a precursor to a wide variety of flavonoids pivotal for plant resilience against both biotic and abiotic environmental stressors [70]. In particular, phenylpropanoids have a deterrent effect on insects, serving as key defense mechanisms in plants against insect herbivore attacks [71]. Interestingly, we found that the treatment with 3-methylbutanol significantly augmented the abundance of the ion signal corresponding to 4-coumaric acid compared to control. Numerous studies underscore the physiological effects of 3-methylbutanol in plants, highlighting its activity as a potent compound capable of eliciting responses on its own [72,73,74,75]. Therefore, our results suggest that the combined influence of VOCs emitted by *B. bassiana* and 3-methylbutanol may trigger the accumulation of phenylpropanoids in plants, consequently affecting the feeding behavior of *S. frugiperda* larvae. 

Entomopathogenic fungi exert a significant selective pressure over various arthropod taxa. The fungal VOC emissions and their role in modulating the feeding behavior of insect herbivores represent evolutionarily favored traits. Hence, we are currently investigating other species of entomopathogenic fungi to compare the efficacy of these volatile molecules. In particular, our study helped elucidate that *S. frugiperda* is capable of perceiving and responding to the presence of *B. bassiana* based on the virulence of the strains, both in larval and adult stages [11]. However, the mechanism by which the insects respond to these fungal VOCs, as well as the breadth of insect taxa they may influence, requires further investigations to determine the ecological implications of these compounds at the ecosystem level. 

## 5. Conclusions

VOCs emitted by the fungal entomopathogen *B. bassiana* influenced the pattern of herbivory in *S. frugiperda* larvae. Furthermore, the larvae exhibited a stronger response when perceiving the most virulent strain. Multiple neurophysiological and innate responses are likely to be activated in insects during exposure to fungal VOCs, warranting further investigation. The modulation of the plant metabolome by *B. bassiana* provides an additional layer of complexity to the interaction between the fungus and the insect. Hence, plant phenolic compounds may play an important role in this interaction. Our results provide a new perspective on the chemical interactions between insect herbivores and fungal entomopathogens in association with plants via VOC emission. Additionally, our study offers an insight into the role of fusel alcohols such as 3-methylbutanol that act as semiochemicals modulating different kinds of insect behaviors, which could facilitate their application in insect pest monitoring and biocontrol programs.

## Figures and Tables

**Table 1:** Ion pattern fragmentation obtained by DIESI MS/MS for the two ions with the highest Gini index.

Putative Compound	Monoisotopic Mass (Da)	UPLC-MS Observed [M+H]^+^	DIESI-MS/MSObserved [M+H]^+^	Fragmentation Patterns (*m*/*z*)	References
4-Coumaric acid	164.047	165.882	164.980	147.187, 146.847, 123.007, 119.005	[45]
Unknown	Unknown	437.402	437.275	418.825, 371.258, 278.793, 214.003	[46,47]

## Data Availability

The original contributions presented in the study are included in the article, further inquiries can be directed to the corresponding authors.
